# Loss of NEDD4 causes complete XY gonadal sex reversal in mice

**DOI:** 10.1038/s41419-022-04519-z

**Published:** 2022-01-24

**Authors:** Simon P. Windley, Chloé Mayère, Alice E. McGovern, Natasha L. Harvey, Serge Nef, Quenten Schwarz, Sharad Kumar, Dagmar Wilhelm

**Affiliations:** 1 Department of Anatomy & Physiology, The University of Melbourne, Parkville, 3010 Australia; 2 Department of Genetic Medicine and Development, University of Geneva, 1211 Geneva, Switzerland; 3 Centre for Cancer Biology, University of South Australia, Adelaide, 5001 Australia

**Keywords:** Disease model, Cell lineage

## Abstract

Gonadogenesis is the process wherein two morphologically distinct organs, the testis and the ovary, arise from a common precursor. In mammals, maleness is driven by the expression of *Sry*. SRY subsequently upregulates the related family member *Sox9* which is responsible for initiating testis differentiation while repressing factors critical to ovarian development such as FOXL2 and β-catenin. Here, we report a hitherto uncharacterised role for the ubiquitin-protein ligase NEDD4 in this process. XY *Nedd4*-deficient mice exhibit complete male-to-female gonadal sex reversal shown by the ectopic upregulation of *Foxl2* expression at the time of gonadal sex determination as well as insufficient upregulation of *Sox9*. This sex reversal extends to germ cells with ectopic expression of SYCP3 in XY *Nedd4-/-* germ cells and significantly higher *Sycp3* transcripts in XY and XX *Nedd4-*deficient mice when compared to both XY and XX controls. Further, *Nedd4-/*- mice exhibit reduced gonadal precursor cell formation and gonadal size as a result of reduced proliferation within the developing gonad as well as reduced *Nr5a1* expression. Together, these results establish an essential role for NEDD4 in XY gonadal sex determination and development and suggest a potential role for NEDD4 in orchestrating these cell fate decisions through the suppression of the female pathway to ensure proper testis differentiation.

## Introduction

Gonadogenesis is a unique process wherein two morphologically distinct organs, the testis and the ovary, arise from a common precursor. Established on the ventromedial side of the mesonephros, this shared anlage develops as a thickening of the epithelium, which proliferates to give rise to the bipotential genital ridges. This process begins as early as 10.0 days post coitum (dpc) in mice with the expression of GATA4, which is quickly followed by expression of nuclear receptor subfamily 5 group A member 1, *Nr5a1* (ref. [1]).

Maleness is determined upon expression of the sex-determining region on the Y chromosome (*Sry*), which is expressed in Sertoli cell precursors starting in the centre of the gonad at 10.5 dpc in mice, before spreading in a centre-to-pole fashion. *Sry* expression is fleeting, however, with peak expression observed at 11.5 dpc, before being downregulated, and becoming all but absent by 12.5 dpc, just 24 hours later [2, 3]. Following this, SRY upregulates the SRY-box containing gene 9 (*Sox9*) and the testis programme is initiated [4]. SRY and SOX9 are both necessary and sufficient in determining the male fate with ablation of these factors resulting in male-to-female sex reversal in XY mice [5, 6] and ectopic expression in XX gonads leading to female-to-male sex reversal [7, 8].

Antagonism between testicular and ovarian promoting pathways exists and as such the testicular phenotype must be actively maintained in the XY gonad while simultaneously repressing pro-ovarian pathways. In this way, ectopic strengthening of the ovarian pathway in XY gonads can result in male-to-female sex reversal as seen in mice over-expressing the pro-ovarian transcription factors FOXL2 (ref. [9]) and CTNNB1 (beta-catenin) [10], owing to the ability of these factors to suppress the testis programme [10–12]. While many factors critical to this process have been identified, the molecular mechanisms driving mammalian sex determination and gonadal development remain incompletely understood. Indeed, while our knowledge of sex determination and gonad development is biased towards the roles of transcription factors and signalling cascades [13] an essential role for post-translational modifications in these processes is becoming increasingly apparent [14–16].

One such modification, ubiquitination, is a process in which the 8.5 kilo Dalton protein ubiquitin is covalently added to protein substrates, enabling the regulation of protein stability and function [17]. The neural precursor cell expressed developmentally downregulated protein 4 (NEDD4) is a ubiquitin-protein ligase and the founding member of the NEDD4 family of HECT type ubiquitin-protein ligases, responsible for the transfer of ubiquitin onto protein substrates [18–20]. NEDD4 has been implicated in a wide array of developmental processes with loss of *Nedd4* in mice resulting in embryonic lethality and growth restriction [21], abnormal cranial neural crest development [22], and perturbations to the developing cardiovascular, nervous and immune systems [23–25].

We have previously shown NEDD4 to be expressed at the time of murine testis differentiation and have identified potential substrates for NEDD4 in the developing testis [26]. Here, we report a role for NEDD4 in the developing gonads by characterising the gonadal phenotype of *Nedd4-*deficient mice. Our results reveal an uncharacterised role for NEDD4 in mouse development with *Nedd4*-deficient mice exhibiting complete male-to-female gonadal sex reversal.

## Results

### Homozygous deletion of Nedd4 causes complete male-to-female gonadal sex reversal

**Fig. 1:**
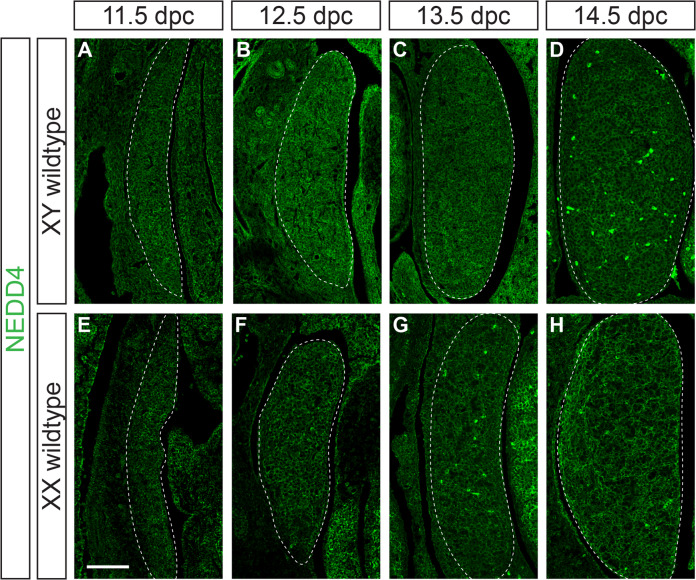
NEDD4 is ubiquitously expressed in the developing XY gonad. Section immunofluorescence on XY (**A**–**D**) and XX (**E**–**H**) wildtype embryos from 11.5 dpc to 14.5 dpc stained for NEDD4 (green). Gonads are denoted by a white dotted line. The anterior pole of each gonad is positioned at the top of each panel. Scale bars = 100 μm.

We have previously shown that NEDD4 is expressed in all cells of the developing testis at 15.5 dpc, with its transcript detectable in both somatic and germ cells from 12.5 to 15.5 dpc [26]. To extend this analysis and show in more detail the expression of NEDD4 protein during gonad differentiation, we performed immunofluorescence on paraffin sections of XX and XY mouse embryonic gonads from 11.5 dpc, the time of gonadal sex determination, to 14.5 dpc, the time at which gonadal sex has become apparent. This analysis showed that NEDD4 is expressed ubiquitously throughout the developing gonads of both sexes at all time points analysed (Fig. 1) and, as expected, no NEDD4 was detected in *Nedd4*-deficient (*Nedd4-/*-) mouse embryos (Supplementary Fig. S1).

**Fig. 2:**
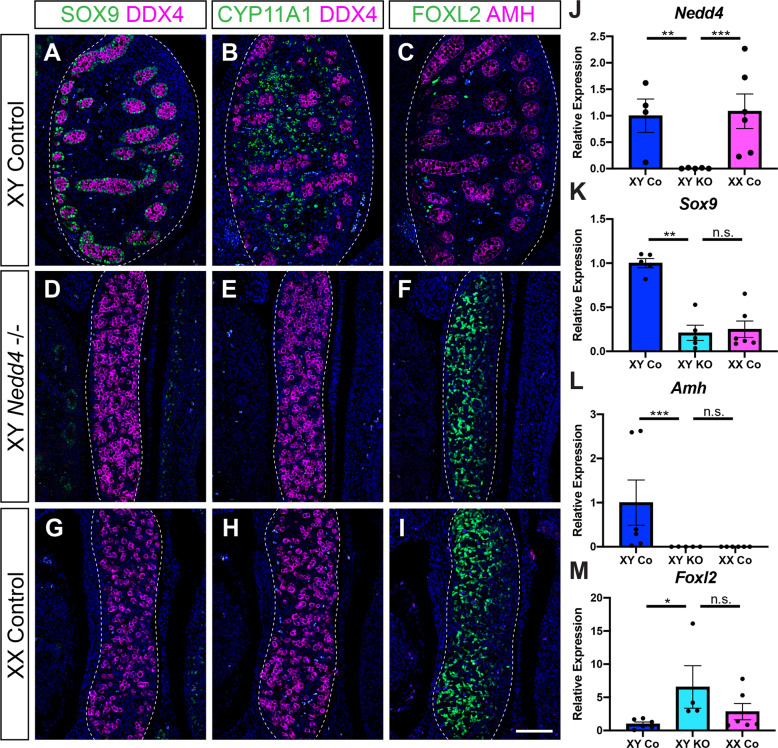
Complete male-to-female sex reversal in XY *Nedd4*-/- mice. **A**–**I** Section immunofluorescence on 14.5 dpc XY *Nedd4-/*- embryos alongside XY and XX littermate controls stained for Sertoli cell markers SOX9 (green in **A**, **D**, **G**) and AMH (magenta in **C**, **F**, **I**), Leydig cell marker CYP11A1 (green in **B**, **E**, **H**), germ cell marker DDX4 (magenta in **A**, **B**, **D**, **E**, **G**, **H**) and granulosa cell marker FOXL2 (green in **C**, **F**, **I**). Gonads are denoted by a white dotted line. The anterior pole of each gonad is positioned at the top of each panel. Scale bars = 100 μm. RT-qPCR analyses of *Nedd4* (**J**), *Sox9* (**K**), *Amh* (**L**) and *Foxl2* (**M**) expression at 14.5 dpc on XY *Nedd4-/*- gonads (KO) (*n* = 5), XY controls (Co) (*n* = 6) and XX controls (*n* = 6). Values are expressed relative to the XY controls. Mean ± SEM; *t*-test, n.s. not significant, *p < 0.05, **p < 0.01, ***p < 0.001.

Next, to characterise the role of *Nedd4* in foetal gonads, we analysed gonads of *Nedd4-/*- at 14.5 dpc, a stage at which disruptions to gonadal sex determination and gonad differentiation are easily detectable by section immunofluorescence for testicular and ovarian markers. As expected, testes of XY littermate controls were round in appearance and showed DDX4 positive germ cells partitioned into testis cords throughout the gonad (Fig. 2A, B). These testis cords also featured Sertoli cells, the testicular supporting cell lineage, as evidenced by the expression of SOX9 (Fig. 2A) and its downstream target AMH (Fig. 2C). CYP11A1 positive steroidogenic Leydig cells were present throughout the interstitium of XY control gonads (Fig. 2B) and FOXL2 positive granulosa cells, the ovarian supporting cell lineage, were all but absent (Fig. 2C). In contrast, XX control gonads were elongated with DDX4 positive cells located throughout the gonad (Fig. 2G, H). XX control gonads showed a lack of SOX9 and AMH positive Sertoli cells (Fig. 2G, I) and CYP11A1 positive interstitial Leydig cells (Fig. 2H) but had FOXL2 positive granulosa cells throughout the developing gonad (Fig. 2I), as expected. Analysis of XY *Nedd4-/-* gonads revealed no apparent testicular structures and were nearly indistinguishable from XX controls (Fig. 2D–I). XY *Nedd4*-deficient gonads did not express the Sertoli cell markers SOX9 and AMH (Fig. 2D, F) nor the Leydig cell marker CYP11A1 (Fig. 2E). Instead, the ovarian granulosa cell marker FOXL2 was expressed throughout XY *Nedd4*-/- gonads (Fig. 2F) indicating that by 14.5 dpc XY *Nedd4-/-* mice exhibit complete male-to-female gonadal sex reversal. This was confirmed at the transcript level using quantitative RT-PCR (RT-qPCR), which showed that XY *Nedd4-/-* gonads had a complete loss of *Nedd4* expression (Fig. 2J), a significant reduction in *Sox9* and *Amh* (Fig. 2K, L) and a significant increase in *Foxl2* (Fig. 2M) relative to XY controls, consistent with ovarian development at this time.

**Fig. 3:**
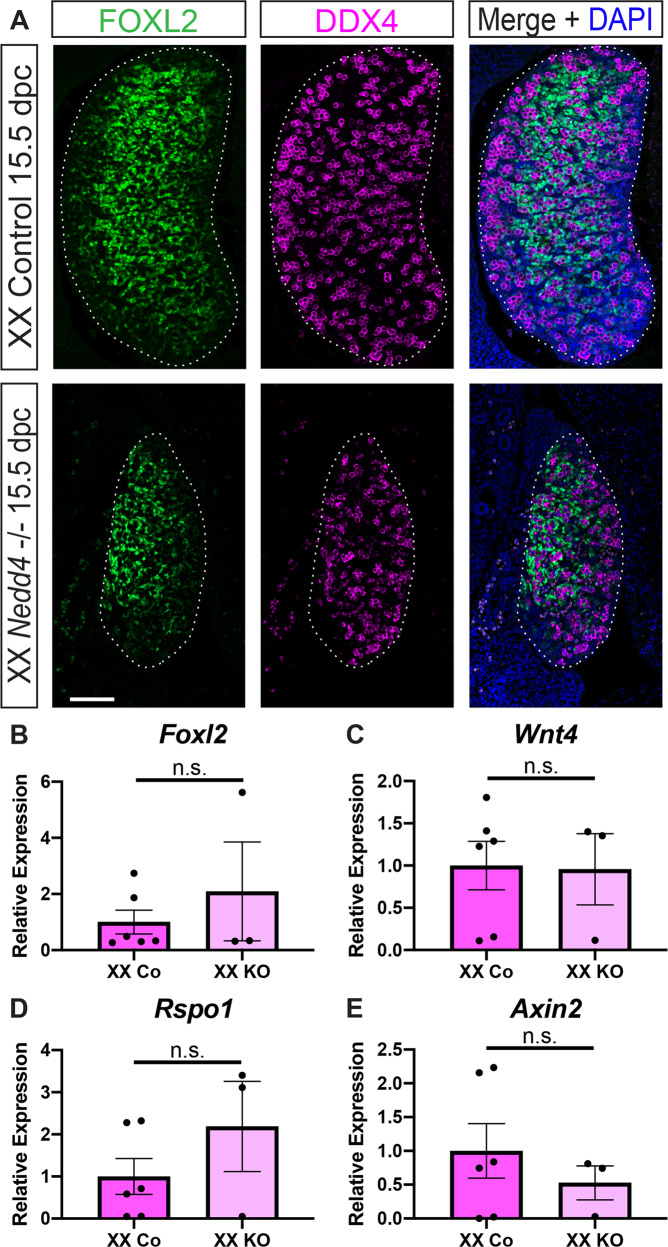
Normal ovarian somatic cell development in XX *Nedd4-/-* mice. **A** Section immunofluorescence on 15.5 dpc XX *Nedd4-/-* embryos alongside XX littermate controls stained for ovarian somatic cells markers FOXL2 (green) and germ cell marker DDX4 (magenta). The anterior pole of each gonad is positioned at the top of each panel. Gonads are denoted by a white dotted line. Scale bars = 100 μm. (**B**–**E**) RT-qPCR analyses of *Foxl2* (**B**), *Wnt4* (**C**), *Rspo1* (**D**) and *Axin2* (**E**) expression at 14.5 dpc on XX *Nedd4-/*- gonads (KO) (*n* = 3) and XX controls (*n* = 6). Values are expressed relative to the XX controls. Mean ± SEM; *t*-test, n.s. not significant.

XX *Nedd4-/-* gonads appeared smaller than XX controls (Fig. 3), however despite the apparent size difference, XX *Nedd4-/-* gonads differentiated as expected, with FOXL2 positive somatic cells and DDX4 positive germ cells present in knockout gonads at 15.5 dpc (Fig. 3A). Further, RT-qPCR showed that *Foxl2, Wnt4, Rspo1* and *Axin2* transcript levels were unchanged in XX *Nedd4-/*- gonads at 14.5 dpc (Fig. 3B–E), indicating that ovarian development proceeds normally in the absence of *Nedd4*.

**Fig. 4:**
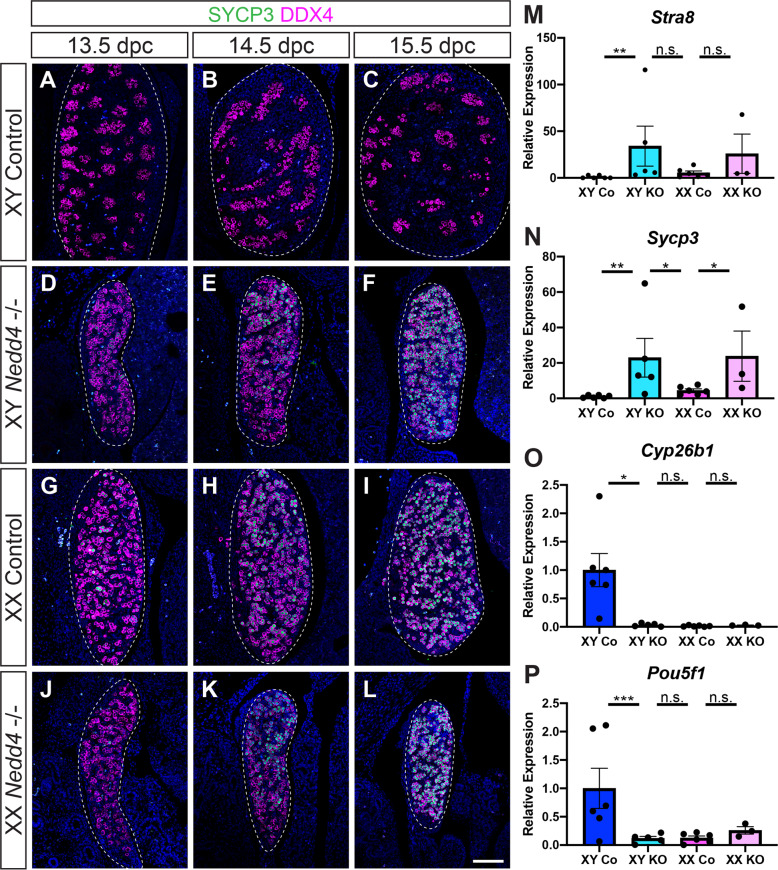
Germ cells in XX *Nedd4*-/- mice enter meiosis. **A**–**L** Section immunofluorescence on 13.5 dpc, 14.5 dpc and 15.5 dpc XY and XX *Nedd4-/-* embryos alongside XY and XX littermate controls stained for germ cell marker DDX4 (magenta) and meiosis marker SYCP3 (green). Gonads are denoted by a white dotted line. The anterior pole of each gonad is positioned at the top of each panel. Scale bars = 100 μm. RT-qPCR analyses of *Stra8* (**M**), *Sycp3* (**N**), *Cyp26b1* (**O**) and *Pou5f1* (**P**) expression at 14.5 dpc on XY *Nedd4-/-* gonads (XY KO) (*n* = 5), XX KO gonads (*n* = 3), XY controls (Co) (*n* = 6) and XX controls (*n* = 6). Values are expressed relative to the XY controls. Mean ± SEM; *t*-test; n.s. not significant, *p < 0.05, **p < 0.01, ***p < 0.001.

Having shown that gonadal somatic cells are sex-reversed in XY *Nedd4-/*- gonads, we next investigated if this extends to primordial germ cells. A distinct difference in germ cell differentiation in the developing ovary compared to the testis is the down-regulation of pluripotency markers such as *Pou5f1* and the entry into meiotic division beginning at ~13.5 dpc [27, 28]. As expected, section immunofluorescence analysis of embryonic gonads from 13.5 dpc to 15.5 dpc showed that XY control samples remained negative for the meiosis marker synaptonemal complex 3 (SYCP3) at all stages analysed (Fig. 4A–C). In contrast, XX controls expressed SYCP3 in a handful of DDX4 positive germ cells as early as 13.5 dpc, before being expressed in almost all germ cells by 15.5 dpc (Fig. 4G–I).

Immunofluorescence analysis of *Nedd4*-/- embryos revealed that mutant gonads were capable of supporting entry into meiosis, with SYCP3 expression observed in a similar fashion to that seen in XX control gonads for both XY (Fig. 4D–F) and XX (Fig. 4J–L) *Nedd4-/*- embryos. RT-qPCR analysis confirmed these data with expression of the meiosis markers *Stra8* (Fig. 4M) and *Sycp3* (Fig. 4N) significantly higher, and expression of *Cyp26b1* (Fig. 4O), which is expressed in Sertoli cells and is responsible for degrading retinoic acid in the testis at this time, as well as *Pou5f1* (Fig. 4P) significantly lower in XY *Nedd4-/-* compared to XY control gonads. Interestingly, while there was no significant difference in transcript levels of *Stra8, Pou5f1* and *Cyp26b1* between XX controls and *Nedd4-/*- gonads of both genetic sexes (Fig. 4M, O, P), *Sycp3* transcripts were significantly higher in XY and XX knockout gonads than in both XY and XX controls (Fig. 4N), perhaps indicative of a role for NEDD4 in suppressing *Sycp3* and regulating entry into meiosis.

### Delayed onset of the testis programme and ectopic FOXL2 in XY Nedd4-/- gonads

**Fig. 5:**
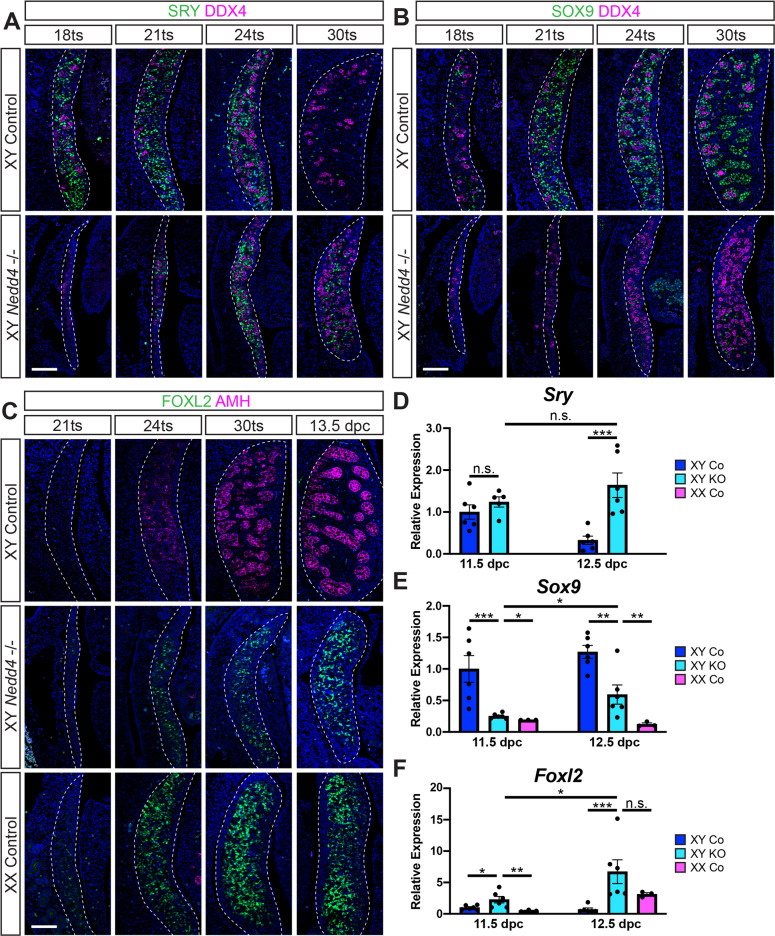
Impaired Sertoli cell differentiation and ectopic FOXL2 in XY *Nedd4*-/- mice. Section immunofluorescence for the male sex determinant SRY (green) (**A**) and Sertoli cell marker SOX9 (green) (**B**) co-labelled with the germ cell marker DDX4 (magenta) on XY *Nedd4-/-* embryos and XY wildtype controls between 18ts and 30ts. **C** Section immunofluorescence for the granulosa cell marker FOXL2 (green) and Sertoli cell marker AMH (magenta) on XY *Nedd4-/-* embryos alongside XY and XX controls between 21ts and 13.5 dpc. The anterior pole of each gonad is positioned at the top of each panel. Gonads are denoted by a white dotted line. Scale bars = 100μm. RT-qPCR analyses of *Sry* (**D**), *Sox9* (**E**) and *Foxl2* (**F**) expression at 11.5 dpc (17–19ts - gonads + mesonephros) and 12.5 dpc (gonads only) on XY *Nedd4-/-* (KO) (light blue; *n* = 6), XY controls (Co) (dark blue; *n* = 6) and XX controls (pink; *n* = 3). Values are expressed relative to 11.5 dpc XY controls. Mean ± SEM; *t*-test; n.s. not significant, *p < 0.05, **p < 0.01, ***p < 0.001.

To determine the basis of the gonadal sex reversal observed in XY *Nedd4-/-* mice at 14.5 dpc, we next analyzed the gonadal phenotype of XY *Nedd4*-deficient mice between 11.5 dpc and 12.5 dpc, the time in which gonadal sex is determined. Morphological analysis of mutant gonads at 11.5 dpc (18 tail somites [ts]) revealed that XY *Nedd4-/*- gonads were substantially smaller than that of their XY control littermates, with the genital ridges appearing only a couple of cell layers thick (Fig. 5A, B).

Immunofluorescence for the testis-determining factor SRY revealed that in XY controls SRY protein was detected throughout the gonads from 18ts (11.5 dpc) to 24ts but was absent at 30ts (12.5 dpc) (Fig. 5A, top panel). In contrast, SRY was not detected in XY *Nedd4-/*- gonads at 18ts but was subsequently expressed in increasing numbers of pre-Sertoli cells from 21ts (~11.75 dpc) to 24ts (12.0 dpc), and was still expressed at 30ts (Fig. 5A, bottom panel), indicating a delay in SRY expression in XY *Nedd4*-deficient mice when compared to XY control littermates.

Interestingly, quantification of *Sry* mRNA revealed no significant difference between XY *Nedd4-/-* mice and XY controls at 11.5 dpc (17–19ts) (Fig. 5D), despite the stark contrast in SRY protein at this stage (Fig. 5A), perhaps indicative of post-transcriptional or post-translational control of the male sex determinant. Consistent with SRY protein expression, however, while *Sry* mRNA was significantly downregulated by 12.5 dpc in XY controls, *Sry* mRNA were maintained in XY *Nedd4*-/- gonads over this time and was significantly higher than *Sry* mRNA in XY control gonads at this stage (Fig. 5D).

We next analysed the expression of SOX9, the principal target of SRY, itself sufficient and necessary for testis differentiation [5, 6, 8]. While SOX9 positive Sertoli cells were apparent in XY controls at 18ts, increased in numbers by 21ts and began to coalesce to form testis cords by 30ts (Fig. 5B, upper panel), SOX9 expression only became detectable in XY *Nedd4-/-* gonads at 24ts and, while the number of SOX9 positive Sertoli cells increased in the 12 hours to 30ts (Fig. 5B, lower panel), this was insufficient to induce the testicular programme in XY *Nedd4-/-* gonads (Fig. 2). Quantification of *Sox9* by RT-qPCR confirmed the lower expression levels, with significantly lower levels of *Sox9* transcript at both 11.5 dpc and 12.5 dpc in XY *Nedd4-/-* compared to XY control gonads, but not as low as XX control gonads (Fig. 5E).

We sought to further characterise the XY sex reversal by analysing the expression of AMH, a known target of SOX9 in the developing testis [29, 30], and FOXL2, the ovarian granulosa cell marker [31, 32] by section immunofluorescence from 21ts to 13.5 dpc (Fig. 5C). While AMH was detected as early as 24ts in XY controls, AMH was absent from XX controls, as expected, and XY *Nedd4-/-* gonads at all stages analysed (Fig. 5C) indicating that the level of SOX9 in *Nedd4* ablated gonads was insufficient to induce the expression of AMH. Similarly, FOXL2 expression in developing XY *Nedd4-/-* gonads was reminiscent of that of XX controls, with few FOXL2 positive cells detectable at 21ts, and subsequently increased in numbers throughout the gonads at later stages (Fig. 5C).

Interestingly, *Foxl2* transcripts were significantly higher in XY *Nedd4-/-* gonads than in both XY and XX controls at 11.5 dpc (Fig. 5F), a potential cause for the gonadal sex reversal later observed in these mice. However, *Foxl2* expression was not significantly higher than in XX control gonads at 12.5 dpc (Fig. 5F). Together, these data suggest that an early failure to sufficiently promote the testis-determining programme during a critical time window, and a concurrent upregulation of the ovary-determining pathway, underlies the sex reversal observed in XY *Nedd4-/-* mice.

### Impaired proliferation and reduced Nr5a1 expression in Nedd4-/- mice

**Fig. 6:**
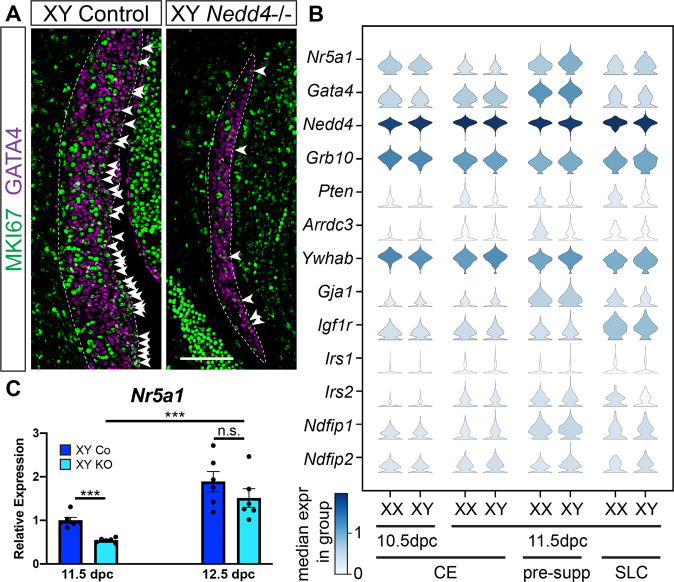
Impaired proliferation and decreased *Nr5a1* expression in XY *Nedd4*-/- mice. **A** Section immunofluorescence on 11.5 dpc (18 tail somites [ts]) XY *Nedd4-/*- embryos and XY controls stained for gonadal somatic cell marker GATA4 (magenta) and proliferation marker MKI67 (green). The anterior pole of each gonad is positioned at the top of each panel. Gonads are denoted by a white dotted line. MKI67-positive cells within the coelomic epithelium are highlighted by white arrowheads. Scale bar = 100μm. **B** Violin plots showing the expression of gonadal progenitor markers *Nr5a1* and *Gata4* as well as *Nedd4* and *Nedd4* interacting proteins in mouse gonadal cells expressing both *Nr5a1* and *Gata4* at E10.5 and E11.5. XX and XY expression levels are shown separately. Expression levels are shown in log-normalised counts. Colour indicates median expression in the group according to the scale shown in the left bottom corner. CE coelomic epithelium, pre-supp pre-supporting cells, SLC supporting-like cells. **C** RT-qPCR analyses of *Nr5a1* at 11.5 dpc (17–19ts, gonads + mesonephroi) and 12.5 dpc (30ts, gonads only) on XY *Nedd4-/*- gonads (KO) (light blue; *n* = 6) and XY controls (Co) (dark blue; *n* = 6). Values are expressed relative to 11.5 dpc XY controls. Mean ± SEM; *t*-test; n.s. not significant, ***p < 0.001.

Given the prevailing size reduction in *Nedd4* mutant gonads, we next analysed the proliferative capacity of the coelomic epithelium, which gives rise to supporting and steroidogenic precursors in the developing gonads [33, 34]. This was achieved by co-immunolabelling mutant and control gonads at 11.5 dpc with the proliferation marker MKI67 and GATA4, the earliest known marker of gonadal somatic cells [1]. We found a marked reduction in the number of proliferative somatic cells (GATA4 positive/MKI67 positive) throughout the genital ridge, including within the coelomic epithelium (Fig. 6A, arrowheads), in XY *Nedd4-/*- mice compared to XY controls. NEDD4 has been shown to control animal growth through the regulation of insulin and IGF1 signalling via the adaptor protein GRB10 [21]. To investigate if the same mechanism could function in the genital ridge, we interrogated our single-cell RNAseq dataset [35] for co-expression of NEDD4 and known interacting proteins including GRB10 [21] in XX and XY coelomic epithelial (CE), pre-supporting and supporting-like cells (SLC [35]), all marked by the expression of *Nr5a1* and *Gata4*, at 10.5 and 11.5 dpc (Fig. 6B). While genes encoding for some of the NEDD4 interacting proteins such as *Pten*, *Arrdc3* and *Irs1* are only expressed at very low levels in these cells, *Nedd4* was co-expressed with *Grb10* and *Igf1r* (Fig. 6B). Given that proliferation has been shown to be an early determinant of the male fate in embryonic gonads [36], defective proliferation of the genital ridges, likely due to inhibition of insulin and/or IGF1 signalling by GRB10, may contribute to the sex reversal observed in *Nedd4-/*- mice.

Finally, given that *Nr5a1* gene expression is significantly reduced in several mouse models displaying XY sex reversal phenotypes [37–40], we assessed whether *Nr5a1* expression was impacted in XY *Nedd4-/-* mice by using RT-qPCR at 11.5 dpc and 12.5 dpc. Indeed, XY *Nedd4-/*- mice showed a near 50% reduction in *Nr5a1* levels at 11.5 dpc (Fig. 6B) which may contribute to the reduction in *Sox9* transcript levels in *Nedd4-/-* mice, owing to the known role of NR5A1 in the promotion of *Sox9* expression [4]. The reduction in *Nr5a1* transcripts was only transient, however, as expression levels were comparable to controls at 12.5 dpc (Fig. 6B).

## Discussion

Here we report an unexpected and novel role for the ubiquitin ligase NEDD4 in mammalian sex determination with *Nedd4-/-* mice exhibiting complete male-to-female gonadal sex reversal. This is likely due to a reduction in gonadal precursor cell proliferation due to regulation of insulin and IGF1 signalling by NEDD4 and its interacting protein GRB10, and therefore an early failure to initiate the testis-determining programme which leads to the ectopic expression of the ovarian transcription factor FOXL2.

Expression of *Nr5a1*, one of the earliest known markers of the developing genital ridges [1], was greatly diminished in gonads of XY *Nedd4-/*- mice (Fig. 6B). This is consistent with previously reported mouse models that displayed reduced *Nr5a1* expression and XY gonadal sex reversal including *Six1/4*, *Insr/Igf1r* and *Cbx2-*deficient mice [38–40]. Similarly, all three of these mouse models exhibited a reduction in proliferation of somatic cell progenitors in-line with the reduction observed in *Nedd4/*- gonads. Given that the *Nr5a1* positive cell population resides in the coelomic epithelium and proliferates to give rise to gonadal somatic cells [33, 41], and perturbations to this process result in XY gonadal sex reversal [36], our results implicate NEDD4 in the formation and proliferation of somatic precursors in the developing gonad. Indeed, NEDD4 has been shown to promote proliferation in a range of biological contexts [42–44] and, as such, it is unsurprising that such a role be ascribed to NEDD4 in the developing gonads. Using a proteomics approach, we recently identified the NEDD4-interactome, including NEDD4 candidate substrates, in a murine testis cell line [26]. The FunRich Biological process analysis of this dataset revealed cell cycle, cell division and positive regulation of cell proliferation and differentiation to be among the top biological processes identified for NEDD4-mediated regulation [26] and, as such, some of these substrates may contribute to the phenotype observed in *Nedd4*-deficient mice. The most likely avenue through which NEDD4 could promote genital ridge proliferation is via modulation of insulin and IGF1 signalling in conjunction with GRB10. Indeed, signalling through IGF1R and IR has been shown to be greatly reduced in *Nedd4-/-* mice, owing to the misregulation of GRB10 [21]. These proteins have been shown to interact in testicular cell lines [26] and we show here that they are co-expressed with NEDD4 in coelomic epithelial, supporting precursor and supporting-like cells in 10.5 and 11.5 dpc genital ridges. Moreover, insulin signalling itself has been shown to be important for testis determination [40], hence it is plausible that a reduction in signalling through IGF1R and IR contributes to the gonadal phenotype observed in these mice.

The reduced proliferation of somatic cells in *Nedd4*-deficient genital ridges likely results in the delay of *Sry* expression [36]. *Sry* must act in a critical time window to promote the testicular fate [45, 46] as first shown by experiments in mice using a combination of a Y chromosome from some *Mus domesticus* subspecies, such as *Mus domesticus poschiavinus* (Y^POS^), with the inbred C57BL/6 genetic background [47], and further defined using a transgenic mouse line with an inducible *Sry* transgene [45]. In these mouse models, a delay in *Sry* expression results in a reduction in *Sox9* levels and subsequent male-to-female sex reversal, which is also observed in XY *Nedd4-/-* gonads. In addition, reduced *Nr5a1* in gonads at this time further compounds the sex reversal observed in these mice, owing to the synergistic roles of SRY and NR5A1 in the promotion of *Sox9* transcription [4]. Furthermore, NEDD4 may also directly regulate the expression of *Sox9*, as *Sox9* transcript and protein levels have been shown to be significantly reduced upon knockdown of *Nedd4* in vitro, albeit in neural crest cell lines [22].

Given the known binding of SOX9 to the *Foxl2* promoter [48] and the ectopic expression of FOXL2 in the absence of *Sox9* [49] it is reasonable to argue that the mere absence of SOX9 is sufficient to explain the ectopic expression of FOXL2 observed in XY *Nedd4-/-* mice. However, if this were the only cause, one may anticipate similar expression levels of *Foxl2* in XY *Nedd4*-/- gonads and XX controls. In contrast, *Foxl2* transcript levels exceed that seen in XX controls, suggesting that the increase in *Foxl2* is partly independent of SOX9 in *Nedd4-/-* gonads. Given that ectopic FOXL2 expression has been shown to repress Sertoli cell differentiation and reduce *Sox9* levels in XY gonads [9, 12] it is possible that this further enforces the sex reversal observed in these mice.

Interestingly, while the gonadal phenotype in XY *Nedd4*-deficient mice mimicked the one in XY gonads from mice with deletion of the insulin receptors, the phenotype in XX gonads did not [40]. In *Insr;Igf1r* mutant gonads ovarian differentiation, including germ cell entry into meiosis, was delayed irrespective of the genetic sex [40]. In contrast, the timing of ovarian differentiation in both XX and XY *Nedd4*-/- mice appeared to be normal. Accordingly, there was no significant difference in the expression of genes associated with entry into meiosis such as *Stra8, Pou5f1* and *Cyp26b1* between gonads from XX wildtype and both XX and XY *Nedd4-/*- mice at 14.5 dpc, further corroborating the notion that ovarian somatic cells differentiated normally, given that the somatic environment drives the development and differentiation of the germline [50, 51]. Surprisingly, expression of the meiotic marker gene *Sycp3* was significantly higher in both XX and XY *Nedd4-/-* gonads when compared to both XX and XY littermate controls. It is unknown, however, whether this is indicative of a germ cell-intrinsic role for NEDD4 or a consequence of the role of NEDD4 in the surrounding somatic cells. Interestingly, NEDD4 has been shown to interact with both NANOS2 and DAZL in spermatogonial stem cells, germ cell derivatives in the postnatal testis [52]. As both NANOS2 and DAZL have been implicated in regulating meiosis and germ cell differentiation during foetal development [53, 54] it is likely that the same interactions between these two proteins and NEDD4 exist in the developing gonads and interrogating the influence of the loss of NEDD4 on these two substrates may prove fruitful in furthering our understanding of the role of this enzyme in the developing germ cells.

Together, our findings highlight an unexpected novel role for the ubiquitin-protein ligase NEDD4 in gonadal sex determination and gonad development, with the loss of *Nedd4* resulting in male-to-female gonadal sex reversal. These results provide possible evidence for a hitherto unrecognised role of this enzyme in human idiopathic XY DSDs.

## Materials and methods

### Mouse lines

*Nedd4-/-* mice, described previously [21], were backcrossed for at least seven generations onto the inbred C57BL/6 strain, obtained from the Animal Resources Centre (Western Australia). *Nedd4-/*- embryos were generated by timed matings of *Nedd4*+*/-* parents, with noon of the day on which a mating plug was observed deemed as 0.5 days post coitum (dpc). For more accurate staging of embryos up to 12.5 dpc, the tail somite stage (ts) was determined by counting the number of somites posterior to the hind limb, with 18ts corresponding to 11.5 dpc, 24ts corresponding to 12.0 dpc and 30ts corresponding to 12.5 dpc [55]. Genotyping at the *Nedd4* locus was performed on genomic DNA derived from tail tissue using the following primer combinations: Nedd4_Geno_WT_F: 5′-*TCTTATGGGTGCTGTGGTTACAG*-3′ and Nedd4_Geno_R: 5′-*CATGTGCTTTACCACTGAGC*-3′ for the wildtype allele and Nedd4_Geno_KO_F: 5′-*CCTTGCAAAATGGCGTTACT*-3′ and Nedd4_Geno_R: 5′-*CATGTGCTTTACCACTGAGC*-3′ for the mutant allele [22]. Genetic sex was determined by PCR as described before [56]. For NEDD4 expression studies, embryos were collected from timed matings of wildtype C57BL/6. All animals obtained that had the required genotype were included in the analyses.

### Immunofluorescence

Section immunofluorescence on paraformaldehyde-fixed, paraffin-embedded mouse embryos was performed as described previously [26]. Each analysis was performed on at least three independent biological samples. Primary antibodies used were rabbit anti-SOX9 (ref. [3]) (1:200), goat anti-DDX4 (R&D Systems, RDSAF2030) (1:300), rabbit anti-CYP11A1 (ref. [57]) (1:300), goat anti-AMH (Santa Cruz, sc6886) (1:300), rabbit anti-FOXL2 (ref. [58]) (1:300), mouse anti-SYCP3 (Abcam, ab97672) (1:100), rabbit anti-SRY [3] (1:100), goat anti-GATA4 (Santa Cruz, sc1237) (1:300), mouse anti-Ki67 (BD Transduction Lab, 550609) (1:100) and mouse anti-NEDD4 (BD Transduction Laboratories, 611481) (1:100). All secondary antibodies were purchased from Invitrogen and were used at a dilution of 1:300. These include donkey anti-mouse Alexa 488 (A21202), donkey anti-rabbit Alexa 488 (A21202), donkey anti-goat 546 (A11056) and donkey anti-mouse Alexa 647 (A31571).

### Quantitative real-time RT-PCR (RT-qPCR)

Embryonic gonads were dissected from the underlying mesonephros for all embryonic stages, except 11.5 dpc where the complex remained intact, and snap-frozen in liquid nitrogen. Total RNA was isolated using Trizol (Ambion) and single-stranded cDNA synthesised using the Protoscript II First Strand cDNA Synthesis Kit (New England Biolabs). qPCR was performed with the SensiFAST SYBR No-ROX Kit (Bioline) using the Rotor-Gene 3000 system (Qiagen). Primers used can be found in Supplementary Table 1. Relative mRNA levels were normalised to *Sdha* and are shown relative to controls as indicated in the Figure legends. Each reaction was performed in technical triplicate and each experiment was performed with at least three biological replicates. Error bars represent the standard error of the mean (SEM) between biological replicates. Statistical significance between groups was determined using a two-tailed, unpaired Student’s *t*-test.

### Single-cell RNAseq analysis

Single-cell isolation, collection, sequencing, data preprocessing and annotation were performed as described [35]. Cells annotated as coelomic epithelium, early supporting-like cells and pre-supporting cells from 10.5 and 11.5 dpc were isolated and a Leiden clustering was applied with 0.2 resolution. Five clusters were obtained and one cluster with a mean expression of *Nr5a1* and *Gata4* below 0.1 was excluded. In total 7407 cells were kept.

## Supplementary information

Supplementary Figure legend and table

Supplementary Figure S1

checklist

## Data Availability

The datasets generated and/or analysed during the current study are available from the corresponding author on reasonable request.
