# Supplementary material for: Loss of NEDD4 causes complete XY gonadal sex reversal in mice
Source: Cell Death Dis. 2022 Jan 24;13(1):75. doi: 10.1038/s41419-022-04519-z (PMC8786929; doi:10.1038/s41419-022-04519-z)
Supplement: Supplementary file 1 — Supplementary Figure legend and table [file 41419_2022_4519_MOESM1_ESM.docx]

**Supplementary material**

**Figure S1. NEDD4 is not expressed in the *Nedd4*-/- XY gonad**Section immunofluorescence on XY control and *Nedd4*-/- embryos at 12.5 dpc stained for NEDD4 (green), DDX4 (magenta) and DAPI. Gonads are denoted by a white dotted line. The anterior pole of each gonad is positioned at the top of each panel. Scale bars = 50 μm.

**Supplementary Table S1: RT-qPCR primer sequences**

| Gene | Primer sequence |
| --- | --- |
| *Sdha* | Sdha_F: 5’- TGTTCAGTTCCACCCCACA -3’  Sdha_R: 5’- TCTCCACGACACCCTTCTGT -3’ |
| *Nedd4* | mNedd4_qPCR_F: 5’- GCACGACATGGAGTCCGT -3’  mNedd4_qPCR_R: 5’- GCTGATGTGTCTGTCCAAAA -3’ |
| *Sox9* | Sox9_F: 5’- GCTCTGGAGGCTGCTGAA -3’  Sox9_R: 5’- CGGGGCTCCTACTTGTAAT -3’ |
| *Amh* | Amh_F: 5’- CGAGCTCTTGCTGAAGTTCCA -3’  Amh_R: 5’- GAAGTCCACGGTTAGCACCAA -3’ |
| *Foxl2* | Foxl2_F: 5’- CGGCATCTACCAGTACATCATAGC -3’  Foxl2_R: 5’- GCACTCGTTGAGGCTGAGGTTG -3’ |
| *Wnt4* | Wnt4_F: 5′- CTGGACTCCCTCCCTGTCTT -3′ Wnt4_R: 5′- ATGCCCTTGTCACTGCAAA -3′ |
| *Rspo1* | Rspo1_F: 5′- CGACATGAACAAATGCATCA -3′ Rspo1_R: 5′- CTCCTGACACTTGGTGCAGA -3′ |
| *Axin2* | Axin2_F: 5′- GCAGGAGCCTCACCCTTC -3′  Axin2_R: 5′- TGCCAGTTTCTTTGGCTCTT -3′ |
| *Stra8* | Stra8_F: 5’- CCTAAGGAAGGCAGTTTACTCCCAGTC -3’  Stra8_R: 5’- GCAGGTTGAAGGATGCTTTGAGC -3’ |
| *Sycp3* | Sycp3_F: 5’- AAATCTGGGAAGCCACCTTTGG -3’  Sycp3_R: 5’- TGGAGCCTTTTCATCAGCAACATC -3’ |
| *Cyp26b1* | Cyp26B1_F: 5’- TGGACTGTGTCATCAAGGAGGT -3’  Cyp26B1_R: 5’- GTCGTGAGTGTCTCGGATGCTA -3’ |
| *Pou5f1* | Pou5f1_F: 5’- TCACCTTGGGGTACACCCAG -3’  Pou5f1_R: 5’- CATGTTCTTAAGGCTGAGCTGC -3’ |
| *Nr5a1* | Nr5a1_qPCR_F:  5’- CGACAAGACGCAGCGTAAG -3’  Nr5a1_qPCR_R:  5’- GATCAGCACGCACAGCTTC -3’ |
