# Supplementary figures and images for: Loss of NEDD4 causes complete XY gonadal sex reversal in mice
Source: Cell Death Dis. 2022 Jan 24;13(1):75. doi: 10.1038/s41419-022-04519-z (PMC8786929; doi:10.1038/s41419-022-04519-z)

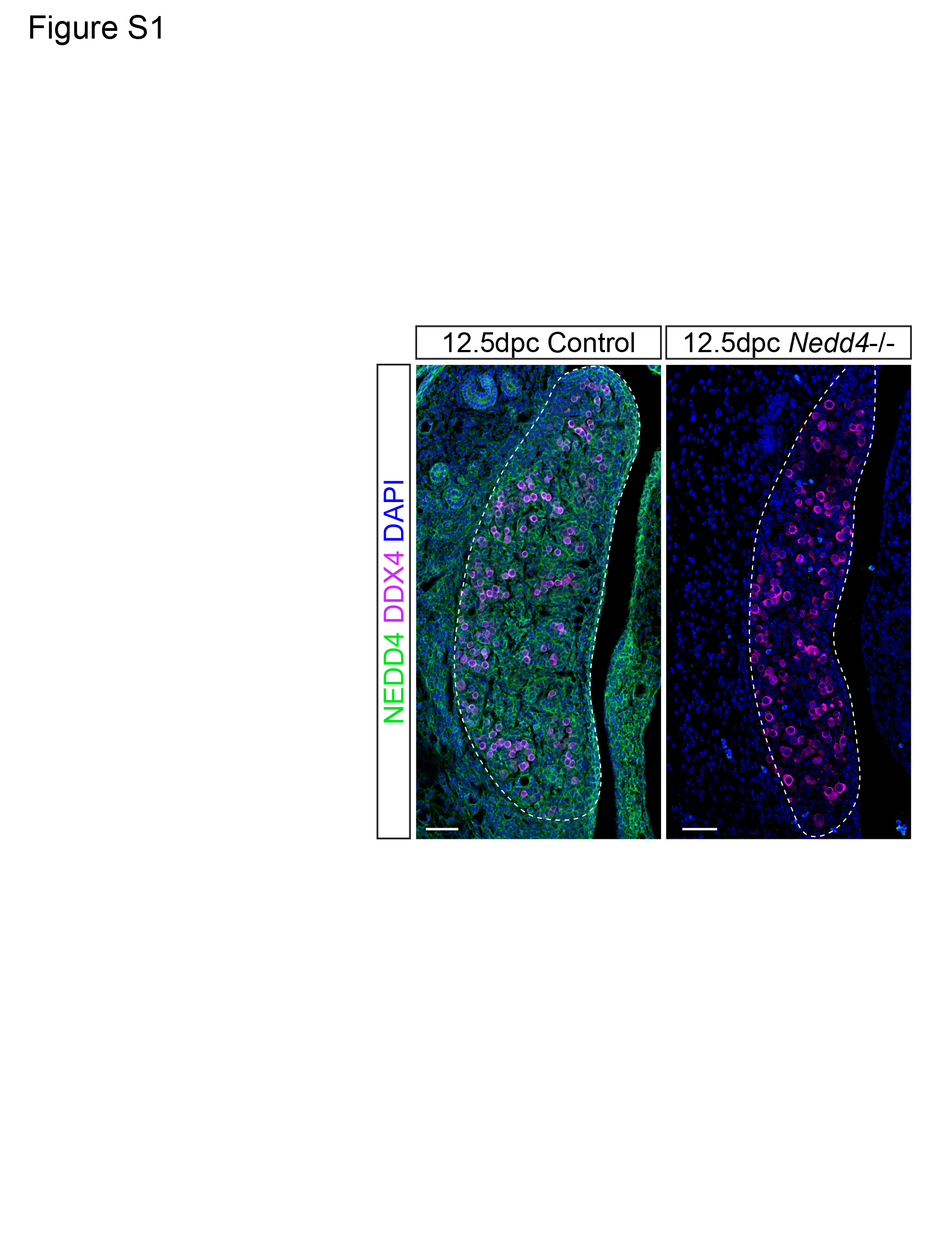

Supplement: Supplementary file 2 — Supplementary Figure S1 [file 41419_2022_4519_MOESM2_ESM.tif]
